# Complete Genome Sequence of Salmonella enterica Serovar Typhimurium Siphophage Skate

**DOI:** 10.1128/MRA.00541-19

**Published:** 2019-07-03

**Authors:** Matthew Rohren, Yicheng Xie, Chandler O’Leary, Rohit Kongari, Jason Gill, Mei Liu

**Affiliations:** a Center for Phage Technology, Texas A&M University, College Station, Texas, USA; Loyola University Chicago

## Abstract

Salmonella enterica serovar Typhimurium is a Gram-negative pathogen and a primary cause of foodborne illnesses worldwide. Here, we present the complete 47,393-bp genome sequence of the siphophage Skate, which was isolated against S. Typhimurium strain LT2.

## ANNOUNCEMENT

Salmonella enterica serovar Typhimurium is a Gram-negative pathogen and a primary cause of foodborne illnesses worldwide (1). S. Typhimurium causes acute inflammatory diarrhea that can progress to invasive systemic disease (2), with at least 400 deaths occurring due to acute salmonellosis every year in the United States alone (3). As a control measure for *S.* Typhimurium, bacteriophages have garnered interest in recent years (4, 5).

The phage Skate was isolated from soil in the cattle holding pen of a cattle harvesting facility in Michigan in August 2016 using *S.* Typhimurium strain LT2 (6) as the host. Host bacteria were cultured on tryptic soy broth or agar (Difco) at 37°C with aeration. Phages were cultured and propagated by the soft agar overlay method (7). The phage was identified as a siphophage using negative-stain transmission electron microscopy performed at the Texas A&M University Microscopy and Imaging Center, as previously described (8). Phage genomic DNA was prepared using a modified Promega Wizard DNA cleanup kit protocol, as described previously (9). Pooled indexed DNA libraries were prepared using the Illumina TruSeq Nano LT kit, and the sequence was obtained from the Illumina MiSeq platform using the MiSeq V2 500-cycle reagent kit (2 × 250-bp reads), following the manufacturer’s instructions, producing 374,842 reads for the index containing the phage genome. FastQC 0.11.5 (https://www.bioinformatics.babraham.ac.uk/projects/fastqc/) was used for quality control of the reads. The reads were trimmed with FASTX-Toolkit 0.0.14 (http://hannonlab.cshl.edu/fastx_toolkit/download.html) before being assembled into a single contig at 160.9-fold coverage using SPAdes 3.5.0 (10). Contig completion was confirmed by PCR using primers (5′-GTCGAAGCGCTACGTGAATA-3′ and 5′-CTTCCCAGAGAGTCCTTTGATAC-3′) facing off the ends of the assembled contig and Sanger sequencing of the resulting product, with the contig sequence manually corrected to match the resulting Sanger sequencing reads. GLIMMER 3.0 (11) and MetaGeneAnnotator 1.0 (12) were used to predict protein-coding genes with manual correction for appropriate gene starts, and tRNA genes were predicted with ARAGORN 2.36 (13). Rho-independent termination sites were identified with TransTerm (http://transterm.cbcb.umd.edu/). Sequence similarity searches by BLASTp 2.2.28 (14) and conserved domain searches with InterProScan 5.15-54.0 (15) were used to predict protein function. All analyses were conducted using default settings via the CPT Galaxy (16) and Web Apollo (17) interfaces (https://cpt.tamu.edu/).

The Skate genome was assembled into a complete contig of 47,393 bp at 160.9-fold coverage. It has a GC content of 46%. Genes coding for proteins involved in morphogenesis, such as the major coat, capsid decoration, tail tube, tail spike, terminase large subunit, and portal proteins, were identified. A lysis cassette consisting of a class II holin, a transglycosylase-type endolysin, and an embedded i-spanin and o-spanin pair were also identified. Genes linked to DNA replication, such as DNA primase, single-stranded DNA binding protein, DNA polymerase subunit, and an ATP-dependent helicase were found. Skate is highly similar to other *Salmonella* phages, like phage IME207 (GenBank accession number KX523699) and E1 (GenBank accession number AM491472) (18, 19), sharing 58% and 55% nucleotide identity, respectively, as determined by progressiveMauve (version 2.4.0) (20).

### Data availability.

The genome sequence of phage Skate was submitted to GenBank as accession number MH321493. The associated BioProject, SRA, and BioSample accession numbers are PRJNA222858, SRR8787571, and SAMN11259649, respectively.
